# Spliceosomal Protein Gene *BmSPX* Regulates Reproductive Organ Development in *Bombyx mori*

**DOI:** 10.3390/ijms21072579

**Published:** 2020-04-08

**Authors:** Yao Wang, Juan Li, Qiu-Xing Wan, Qin Zhao, Kai-Xuan Wang, Xing-Fu Zha

**Affiliations:** 1 State Key Laboratory of Silkworm Genome Biology, Biological Science Research Center, Southwest University, Beibei, Chongqing 400715, China; tifa1224@163.com (Y.W.); worker@swu.edu.cn (J.L.); Wanqiux@outlook.com (Q.-X.W.); 15516767013@163.com (Q.Z.); wkx7418@email.swu.edu.cn (K.-X.W.); 2 Chongqing Key Laboratory of Sericultural Science, Southwest University, Chongqing 400715, China; 3 Chongqing Engineering and Technology Research Center for Novel Silk Materials, Southwest University, Chongqing 400715, China; 4 The State Key Laboratory of Genetic Engineering and MOE Key Laboratory of Contemporary Anthropology, School of Life Science, Fudan University, Shanghai 200438, China

**Keywords:** BmSPX, BmPSI, *Bmdsx*, sex determination, silkworm, transgene

## Abstract

Sex determination and differentiation are nearly universal to all eukaryotic organisms, encompassing diverse systems and mechanisms. Here, we identified a spliceosomal protein gene *BmSPX* involved in sex determination of the lepidopeteran insect, *Bombyx mori*. In a transgenic silkworm line that overexpressed the *BmSPX* gene, transgenic silkworm males exhibited differences in their external genitalia compared to wild-type males, but normal internal genitalia. Additionally, transgenic silkworm females exhibited a developmental disorder of the reproductive organs. Upregulation of *BmSPX* significantly increased the expression levels of sex-determining genes (*BmMasc* and *BmIMP*) and reduced the female-type splice isoform of *Bmdsx*, which is a key switch gene downstream of the sex-determination pathway. Additionally, co-immunoprecipitation assays confirmed an interaction between the BmSPX protein and BmPSI, an upstream regulatory factor of *Bmdsx*. Quantitative real-time PCR showed that BmSPX over-expression upregulated the expression of the *Hox* gene *abdominal-B* (*Adb-B*), which is required for specification of the posterior abdomen, external genitalia, and gonads of insects, as well as the genes in the Receptor Tyrosine Kinase (RTK) signaling pathway. In conclusion, our study suggested the involvement of BmSPX, identified as a novel regulatory factor, in the sex-determination pathway and regulation of reproductive organ development in silkworms.

## 1. Introduction

Sex determination is a fundamental process, in almost all eukaryotic organisms, that controls morphological, physiological, and behavioral differences between the sexes. However, females and males are determined by diverse mechanisms in many taxa, such as insects [1,2]. The bottom of sex determination systems in insects are conserved, and there are diverse primary signals at the top of the sex determination pathway [3]. The primary signal that triggers sex determination is processed by a cascade of genes, ending with the conserved switch gene doublesex (*dsx*) that controls sexual differentiation. In *Drosophila*, the process is controlled by the primary signal, the number of X chromosomes [4], and a regulatory cascade involving key genes such as *Sex lethal*, *transformer* (*tra*), and *dsx* [5]. In the lepidopteran *Bombyx mori*, a homolog of the *dsx* gene exists, but not of *tra* [6,7]. Therefore, the underlying mechanism of silkworm sex determination is clearly different from that of *Drosophila*.

In insects, sexually dimorphic morphology results from products of the *dsx* gene and sex-specific gene regulation mediated by the HOX protein encoded by *Abdominal-B* (*Abd-B*) [8]. *Abd-B* is also required to specify the posterior abdomen and genitalia [9]. In *Bombyx mori*, the homolog of *dsx* (*Bmdsx*) is alternatively spliced into female- and male-specific mRNA transcripts, with female-specific splicing representing the default mode [6,10]. Studies of transgenic worms showed that *Bmdsx* works as a double-switch gene at the final step in the silkworm sex determination cascade [3,11]. Suzuki et al. developed an in vivo, sex-specific splicing assay system using sexually differentiated cultured cells and found that three distinct sequences (CE1, CE2, and CE3), which are located in *Bmdsx* exon 4, served as splicing silencers of male-specific splicing [12]. A nuclear factor, *Bombyx mori* P-element somatic inhibitor (BmPSI), was shown to bind to the CE1 site and is involved in regulating the sex-specific splicing of *Bmdsx* [13]. Moreover, male-specific BmIMP protein could promote the binding between BmPSI and Bmdsx pre-mRNA to promote male-specific splicing of *Bmdsx* [14,15].

The silkworm uses the ZW sex-determining system, which differs from the XY-type system of *Drosophila*, and it includes heterotypic chromosomes (ZW type) in females and homotypic chromosomes (ZZ type) in males [11,16]. A dominant feminizing factor *Fem* located in the W chromosome determines *B. mori* femaleness [7,17]. *Fem* RNA is transcribed from the W chromosome and produces female-specific PIWI-interacting RNA (piRNA) that targets the mRNA of the protein-coding gene *Masculinizer* (*Masc*) [18,19]. The *Masc* gene encodes a CCCH-type zinc finger protein that controls masculinization in male embryos [15]. A knockdown of *Masc* expression led to the production of the female-specific isoform of *Bmdsx* and a dramatic reduction of *BmIMP* expression in male embryos [13]. Further, transfection of *Masc* cDNA into female-specific cells resulted in male-type splicing of *Bmdsx*. However, the knockout mutation assay showed that loss of *BmIMP* function did not affect sexual differentiation in the silkworm. These previous studies indicate that the regulatory mechanism of *Bmdsx* is not well known, and other factors required for sex-specific splicing of *Bmdsx* may exist [20]. Recently, it was reported that over-expression of RNA-binding protein genes BxRBP1 and BxRBP3 efficiently inhibited female-specific splicing type of *Bmdsx* in female-specific cells and generated the male-specific isoform [21]. To explore more regulatory factors involved in alternative splicing of *Bmdsx*, we constructed a yeast two-hybrid cDNA library of the silkworm early embryo and screened regulatory factors shown to interact with BmPSI in a previous study [22]. The results showed that a spliceosome protein BmSPX could bind to BmPSI. BmSPX is a homolog of a spliceosomal protein on the X chromosome of *Drosophila* (SPX) in the silkworm. However, our knowledge of BmSPX in sex determination is limited.

In this study, we produced a transgenic silkworm line that overexpressed the *BmSPX* gene to determine whether *BmSPX* is involved in sex determination and differentiation in the silkworm.

## 2. Results

### 2.1. Construction of BmSPX Overexpression Transgenic Line and Phenotype

**Figure 1:**
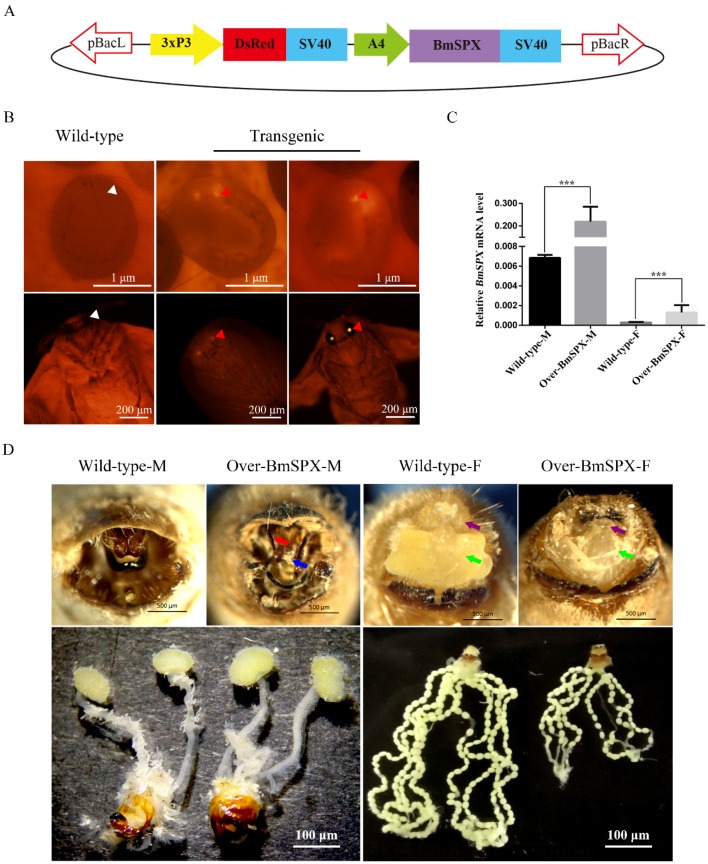
Establishment and phenotypic observation of Over-BmSPX transgenic strains. (**A**) Structure of piggyBac-BmSPX recombinant vector. 3xP3 and A4 promoters were used in the recombinant vector. The SV40 terminator was used to stop the transcription. pBacL and pBacR indicate the left and right terminal inverted repeats. 3xP3 indicate a hyperactive promoter containing three binding sites for Pax-6 homodimers in front of a TATA box. DsRed, *Discosoma* sp. Red Fluorescent Protein. SV40, Terminator of *Simian virus 40*. A4, Actin 4 promotor of *B. mori*. (**B**) The process of screening transgenic strains. The signal of DsRed in transgenic strain is indicated with red triangles. The positions without DsRed in Wild-type strain are indicated with white triangles. (**C**) Real-time PCR of *BmSpx* to confirm *BmSpx* over-expression. A value of *p* < 0.05 was considered to be statistically significant (* *p* < 0.05, ** *p* < 0.01, *** *p* < 0.001). (**D**) Phenotypic and gonad observations of Over-BmSPX strains. Wild-type-M and Wild-type-F indicate the male and female of wild type silkworm, respectively. Over-BmSPX-M and Over-BmSPX-F indicate the male and female of Over-BmSPX transgenic strains, respectively. An unknown thin needle rod in the middle of the gonad in Over-BmSPX-M is indicated with a blue arrow. Two sharp claspers in Over-BmSPX-M are indicated with a red arrow. The developmental disorders of two parts of alluring gland in Over-BmSPX-F are indicated with purple and green arrows.

To determine whether *BmSPX* participates in sex determination of the silkworm, the piggyback-BmSPX vector and helper vector were micro-injected into silkworm embryos (Figure 1A). Through breeding and fluorescent screening of many generations, transgenic lines with high expression of *BmSPX* were obtained and named as Over-BmSPX (Figure 1B). To identify the insertion site of the transgenic lines, we carried out inverse PCR using genomic DNA of the transgenic lines. The PCR products were cloned and sequenced. Sequence analysis showed that transgenic *BmSPX* was located in chromosome 11, and the insert site was located in the intergenic region (Table 1).

To investigate the expression level of *BmSPX* in the transgenic line, Over-BmSPX, we performed Quantitative Real-Time PCR (qPCR) using larvae of Over-BmSPX and wild-type D9L. Compared to the wild-type, *BmSPX* expression was significantly increased in both sexes of the transgenic line at the RNA level (Figure 1C). These results revealed that the transgenic line Over-BmSPX over-expressed *BmSPX*.

When the transgenic lines were reared to moth stage, phenotypic and gonad observation were performed (Figure 1D). In transgenic males, an external genital development disorder was observed, displaying an unknown thin needle rod in the middle of the gonad and sharp claspers, which is more convenient for males to hook the external gonads of female individuals to complete the mating process. There was no obvious difference in male internal genitalia. Further, the transgenic females showed a developmental disorder of their reproductive organs. The external genitalia were defective without a nature alluring gland. For the internal gonads, we found that the ovarian tubes became shorter, the number of eggs decreased, and the size of a single egg became smaller compared to the wild-type silkworm (Figure 1D). These results indicate that *BmSPX* has an effect on the development of reproductive organs in the silkworm.

### 2.2. Over-Expression of BmSPX Influences Expression Level of Key Genes in Sex Determination Pathway

**Figure 2:**
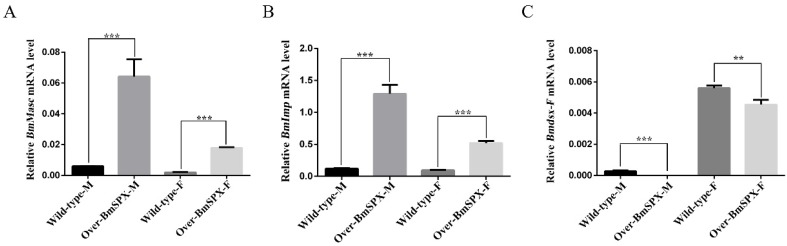
Expression levels of key factors in sex determination of Over-BmSPX transgenic strains. A value of *p* < 0.05 was considered to be statistically significant (* *p* < 0.05, ** *p* < 0.01, *** *p* < 0.001). (**A**) *BmMasc* expression level in Over-BmSPX transgenic strains. (**B**) *BmImp* expression level in Over-BmSPX transgenic strains. (**C**) Expression level of female-specific splicing of *Bmdsx* in Over-BmSPX transgenic strains. Wild-type-M and Wild-type-F indicate the male and female of wild type silkworm, respectively. Over-BmSPX-M and Over-BmSPX-F indicate the male and female of Over-BmSPX transgenic strains, respectively.

According to the phenotypic observation of Over-BmSPX transgenic strains, *BmSpx* was found initially for gonad development. To study the function of *BmSpx* in sex determination of the silkworm, we extracted RNA from transgenic samples for reverse transcription to detect the expression level of key factors in sex determination by qPCR. *Masc* was found as a key regulatory factor, which controls masculinization in male embryos [20]. *BmImp* enhances the male-specific splicing of *Bmdsx* by increasing the binding activity between BmPSI and *Bmdsx* pre-mRNA. The expression levels of *Masc* and *BmImp* were found to be upregulated in both sexes of the transgenic line (Figure 2A,B). *Bmdsx* acts as the double-switch gene at the last step of the sex determination cascade of silkworms. Further, detection of the female-specific splicing isoform of *Bmdsx* (Bmdsx-F) was found to be downregulated in both male and female transgenic silkworms (Figure 2C). These results indicate that BmSPX plays an important role in sex determination of the silkworm.

### 2.3. BmSPX Probably Acts as a Spliceosomal Component to Regulate Alternative Splicing of Bmdsx

**Figure 3:**
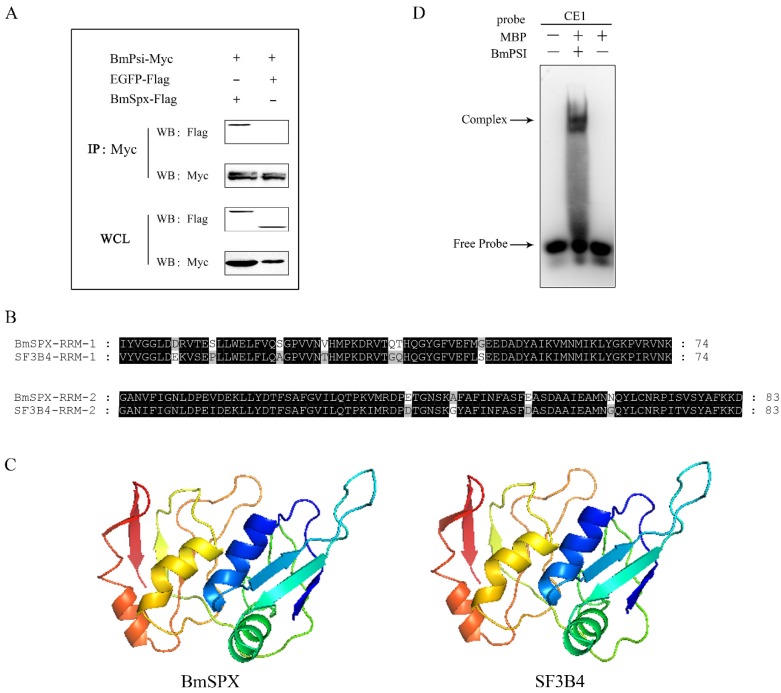
BmSPX may participate in alternative splicing of *Bmdsx* as a splicing factor. (**A**) Co-immunoprecipitation (CO-IP) experiment between BmSPX and BmPSI. IP:Myc indicates that CO-IP experiment was carried out with Myc antibody. WB:Myc and WB:Flag indicate that Western Blot experiments were carried out with Myc antibody and Flag antibody, respectively. WCL, Whole cell lysate. (**B**) Sequence alignment of the RRM domain between BmSPX and its homologous protein SF3B4. RRM, RNA recognition motif. Black and grey indicate identical and similar amino acids, respectively. (**C**) The tertiary structure of BmSPX and SF3B4. (**D**) The electrophoretic mobility shift assay (EMSA) experiment between BmPSI and RNA probe CE1. MBP, Maltose binding protein.

In the previous study, we found BmSPX binding to BmPSI by yeast two-hybrid screening. To confirm the interaction between BmSPX and BmPSI, co-immunoprecipitation (CO-IP) was performed with embryonic cells of silkworm (BmE) in this study. CO-IP demonstrated obvious binding between BmSPX and BmPSI, while there was no binding affinity between BmSPX and enhanced green fluorescent protein (EGFP, as a negative control) (Figure 3A). In addition, the tertiary structure of BmSPX was predicted using a Swiss-model with amino acid sequences (Figure 3B) and showed that BmSPX was similar to splicing factor 3b subunit 4 (Figure 3C), which was reported to be a component of the U2 pre-mRNA spliceosomal complex [23,24]. The two function domains (RRM) of BmSPX and SF3b4 were highly conserved (Figure 3B), and the Swiss-models of these two proteins were almost identical (Figure 3C). In addition, to further confirm the binding between BmPSI and pre-mRNA of Bmdsx, the electrophoretic mobility shift assay (EMSA) experiment between BmPSI and the CE1 RNA probe was performed, which showed that there is an obvious binding between them (Figure 3D). Furthermore, over-expression of *BmSPX* significantly reduced expression of the female-type splice isoform of *Bmdsx* (*Bmdsx-F*), which is regulated by BmPSI (Figure 2C). These results suggested that BmSPX probably functioned as a component of the upstream regulatory complex (spliceosome) to influence alternative splicing of *Bmdsx* [24].

### 2.4. Transgenic Line Over-BmSPX Alters Expression of Genes Involved in Sex Differentiation

**Figure 4:**
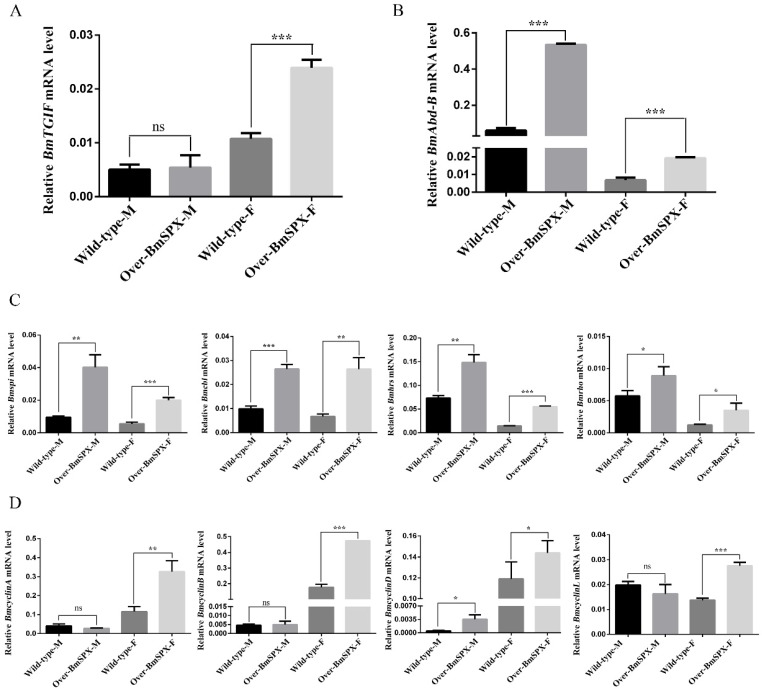
Expression levels of key factors in gonad development of Over-BmSPX transgenic strains. A value of *p* < 0.05 was considered to be statistically significant (* *p* < 0.05, ** *p* < 0.01, *** *p* < 0.001). (**A**) *BmTGIF* expression level in Over-BmSPX transgenic strains. (**B**) *BmAbd-B* expression level in Over-BmSPX transgenic strains. (**C**) Expression level of key factors, including in RTK, in Over-BmSPX transgenic strains. (**D**) Expression level of cyclin genes in Over-BmSPX transgenic strains. Wild-type-M and Wild-type-F indicate the male and female of wild type silkworm, respectively. Over-BmSPX-M and Over-BmSPX-F indicate the male and female of Over-BmSPX transgenic strains, respectively.

Sex differentiation is decided by sex difference genes, which are regulated by the sex-specific splicing of *Bmdsx*. Among these genes, Bombyx mori TGFB-induced factor homeobox (BmTGIF) protein can recruit other factors to form a complex to regulate the genes required for meiotic divisions and spermatid differentiation [25]. The expression level of *BmTGIF* was detected, which showed significant upregulation in female transgenic strains (Figure 4A). The development of insect body segments is related to the *Hox* gene group, including *Abd-B* that is involved in the differentiation of the posterior and terminal parts of insect body segments, which is directly related to differentiation of the external genitalia [8,26]. The expression level of *Abd-B* was significantly increased in both sexes of the transgenic strains (Figure 4B). In the wild-type silkworm, the expression level of *Adb-B* was higher in males than females. However, in the transgenic Over-BmSPX strains, *Adb-B* expression showed significant increases in both sexes, with the tendency of masculinization. Referring to the regulation of *Drosophila dsx* on downstream genes affecting sex differentiation, genes that may be downstream targets of *B. mori dsx* (*Bmdsx*) were detected in the transgenic strains. In the larval stage of silkworm *B. mori*, the epidermis of the middle layer of tissue cells is surrounded by epidermal cells. During gonadal development, there is a difference in the rate of proliferation between the two cell types mediated by *Bmdsx*, which is predicted to be regulated by the RTK signal channel [27]. RTK is mainly composed of Torso, Epidermal growth factor receptor (EGFR), Fibroblast growth factor receptor (FGFR), etc., which can transmit extracellular signals into cells, thereby affecting cell migration and development [28]. The expression levels of key factors in RTK were detected, and their expression levels were increased significantly in transgenic strains (Figure 4C). *Bombyx mori Spitz* (*Bmspi*)*, Bombyx mori Cbl proto-oncogene* (*Bmcbl*)*, Bombyx mori Hepatocyte growth factor-regulated tyrosine kinase substrate* (*Bmhrs*), and *Bombyx mori Rhodopsin* (*Bmrho*) are homologous genes of the key factors in the RTK signaling pathway. These genes showed the same tendency, in which their expression levels in males were higher than females. Moreover, the transgenic strains showed higher expression levels of these genes compared to the wild-type silkworm, which showed a significant masculinization tendency. It was reported that *cyclinD* is the most downstream target gene in the EGFR signaling pathway [29,30]. The expression level of four cyclin genes, *BmcyclinA, BmcyclinB, BmcyclinD,* and *BmcyclinL*, were detected, which were all significantly upregulated in the transgenic female silkworm (Figure 4D).

## 3. Discussion

In this study, we found that the predicted spliceosomal gene *BmSPX* regulated the development of reproductive organs in the silkworm. The spliceosome is a multi-subunit RNA-protein complex involved in the removal of introns from an mRNA precursor. RNA splicing is regulated by two spliceosomes, the major (U2 small nuclear ribonucleoprotein -dependent) and minor (U12 small nuclear ribonucleoprotein -dependent) spliceosomes [31]. U2 small nuclear ribonucleoprotein (snRNP) is composed of U2 snRNA and the splicing factor 3a, 3b (SF3a, SF3b) complex to form the major spliceosome [32]. SF3b4 is a constituent of the SF3b complex in the U2 small nuclear ribonucleoprotein particle. BmSPX is homologous to the splicing factor SF3b4 in the silkworm. In previous studies, SF3b4 was reported to participate in the regulation of the cell cycle, cell differentiation, and the mutation or deletion of the SF3b4 gene, resulting in immunodeficiency and tumorigenesis [32,33]. Since BmSPX and SF3b4 are highly homologous, we assume that BmSPX has an important role in alternative splicing processes in the silkworm. Further, according to our results, BmSPX was shown to be involved in alternative splicing of the key sex-determining switch gene *Bmdsx* via binding to the BmPSI protein. In *Drosophila*, the sex-determining switch gene *dsx* is regulated by *tra* together with *tra2*. However, no homolog of *tra* is present in the silkworm. These results suggested that the upstream regulatory mechanism of *dsx* differs between *B. mori* and *Drosophila*.

BmPSI protein could bind to the pre-mRNA of *Bmdsx* and promote male-specific splicing of *Bmdsx* in the sex determination of *B. mori*. BmSPX was identified to interact with BmPSI through yeast two-hybrid screening [22], and an interaction between them was confirmed by CO-IP in our study. Further, tertiary structure prediction of BmSPX showed that BmSPX has a similar tertiary structure with some key alternative splicing factors in other species. Therefore, we speculated that BmSPX participated in *Bmdsx* splicing.

For further confirmation, the Over-BmSPX transgenic strains were developed, and phenotypic and gonad observation were performed on the transgenic silkworm. The abnormal development of the male external gonads and testicular enlargement in the transgenic strains suggested a reproductive developmental defect in the transgenic silkworm during individual development compared with the wild-type silkworm. Coincidently, the ovarian developmental defects and ovarian tube developmental disorders in transgenic silkworm also showed a reproductive development defect in the female genital organ. The real-time PCR of transgenic strains showed that the upregulation of *BmSpx* caused significant variation in the expression of *Bmdsx* and other key factors of sex determination. The expression levels of *BmMasc* and *BmImp* were significantly upregulated, while the expression of female-specific splicing of *Bmdsx* was sharply downregulated, which all suggested that the alternative splicing of *Bmdsx* tended to be male-specific. A previous study showed that the expression level of BmSpx in testis is obviously higher than every other tissue in fifth-instar day 3, analyzed from genome-wide microarray expression data. Moreover, the over-expression of *BmSPX* in transgenic strains promoted male-specific splicing of *Bmdsx*, resulting in developmental disorders of male and female transgenic silkworms.

To elucidate the mechanism of *BmSpx* regulation, some key regulatory factors of gonad development were detected by real-time PCR. *BmAbd-B* in the *Hox* gene group, which was predicted to regulate differentiation of the terminal parts of silkworms, was found to be sharply increased in both male and female transgenic silkworms. The expression level of *BmAbd-B* in wild-type male silkworms was clearly higher than wild-type female silkworms. In transgenic Over-BmSPX strains, the expression level of *BmAbd-B* showed significant increases in both males and females, which revealed the same as masculinization tendency. *BmTGIF*, that regulates genes required for meiotic divisions and spermatid differentiation, was found to be significantly upregulated in female transgenic silkworms, which may disrupt female homeostasis. *Bmspi*, *Bmcbl*, *Bmhrs*, and *Bmrho* are the homologous genes in silkworms of key genes in the RTK signaling pathway, which showed the same tendency of higher expression in males than in females. The transgenic strains had higher expression levels of these genes compared with wild-type silkworms, which showed a significant masculinization tendency. Finally, it was concluded that the upregulation of *BmSpx* resulted in a masculinization tendency of most parts of the RTK signaling pathway. Dysregulation of the RTK signaling pathway may cause changes in the cell proliferation rate of transgenic strains. The real-time PCR results showed that *BmcyclinA*, *BmcyclinB*, *BmcyclinD*, and *BmcyclinL* genes were expressed abnormally in transgenic silkworms, which corresponded with the gonad development disorder in Over-BmSPX strains. Therefore, changes in *BmSpx* expression levels caused a significant change in *Bmdsx* splicing or other unknown mechanisms to influence sex differentiation of the silkworm.

Our findings suggested that *BmSpx* is a participant in gonadal development, improved the pathway of sex determination, and provided new insights for further research on sex determination in *B. mori*.

## 4. Materials and Methods

### 4.1. Silkworm Strain and Cell

The silkworm non-diapausal strain (*D9L*) was provided from the Gene Resource Library of Domesticated Silkworm, Southwest University in China. The larvae was reared on fresh mulberry leaves at 25 ± 2 °C under a photoperiod of 12 h light/12 h dark with 75% relative humidity. The silkworm cell line BmE (referred to as BmE-SWU1), which was originally developed from embryos, was obtained from the State Key Laboratory of Silkworm Genome Biology. BmE was maintained at 27 °C in Grace’s insect culture medium supplemented with 10% (*v*/*v*) fetal bovine serum (FBS) (Thermo Fisher Scientific, Waltham, MA, USA).

### 4.2. Construction of Recombinant Vectors

The full-length coding sequence (CDS) of the BmSPX gene (GenBank No.: NM_001044181) was amplified by RT-PCR, with the primers designed with restriction enzyme cutting sites (BamH I and Not I) and applicable Flag-Tag sequences (Table 2). The PCR fragment of *BmSPX* was cloned into the pSL1180 (Hr3-BmCP231P-SV40) vector (conserved in our laboratory) to generate the recombinant pSL1180-BmSPX vector. The *BmPSI* gene and enhanced green fluorescent protein (EGFP) were also cloned into the same vector to produce pSL1180-BmPSI and pSL1180-EGFP, respectively. The recombinant pSL1180-BmSPX vector was cut by Asc I. Following, BmSPX with the hr3 enhancer, Actin4 (A4) promoter, and SV40 termination signal, were subcloned into the piggyBac (3xP3-Red-SV40) vector. We verified the sequence of the recombinant vector by sequencing in the Beijing Genomics Institute. The recombinant piggyBac-BmSPX vector was validated and ready for micro-injection.

### 4.3. Establishment of Over-BmSPX Transgenic Strains

The recombinant piggyBac-BmSPX vector and helper DNA were injected into fresh *D9L* silkworm eggs that were laid within four hours, which were then incubated at 25 °C in a humidified chamber for approximately 10 days until hatching. Hatched larvae were bred as generation G_0_, reared to adults under laboratory conditions, and then sib-mated. G_1_ progeny were screened for the presence of the marker gene during the embryonic stage under a fluorescence microscope (Olympus, Tokyo, Japan). Silkworms with positive fluorescence markers were used in subsequent experiments. Genomic DNA was extracted from the *BmSPX* transgenic silkworms, fully digested with Hae III, and self-ligated. Inverse PCR was run to analyze the insertion site using the transposon-specific primers pBacL and pBacR, as described in the previous study [34].

### 4.4. Quantitative Real-Time PCR (qPCR)

Total RNA of negative and positive transgenic strain material was extracted with every whole silkworm using TRIzol^®^ reagent (Invitrogen, Carlsbad, CA, USA), and reverse transcribed using M- Moloney Murine Leukemia Virus (MLV) Reverse Transcriptase (Promega, Madison, WI, USA). qPCR was performed to quantify the RNA levels of the silkworm genes using the SYBR^®^ Premix Ex Taq™ (Tli RNaseH Plus) Kit (TaKaRa, Kusatsu, Japan). The reactions were run on an ABI7500 Real-Time PCR machine (Applied Biosystems, Foster City, CA, USA). The eukaryotic translation initiation factor 4A (silkworm microarray probe ID sw22934) was used as the internal control. The primers used for qPCR are listed in Table 3. The experiment was repeated three times with biological and technical replicates.

### 4.5. Three-Dimensional Structure Prediction of BmSPX

A homology search was carried out using BLAST in the NCBI (National Center for Biotechnology Information) database (non-redundant) using the sequence of the BmSPX protein as a query sequence. The local sequence alignment was performed using ClustalW (http://www.ebi.ac.uk/Tools/msa/clustalw2/). Structure prediction of the BmSPX protein was performed using Swiss-Model, which is an online automated protein structure homology-modeling server (https://swissmodel.expasy.org/interactive). Proteins with similar tertiary structures to BmSPX were found distinctly according to the Swiss-Model analysis.

### 4.6. Co-Immunoprecipitation between BmSPX and BmPSI

As described above, Flag-tagged BmSPX, Myc-tagged BmPSI, and Flag-tagged EGFP coding sequences were cloned into the pSL1180 vector. The cells were transfected with the recombinant plasmids of the experiment and control groups. Cells were collected at 72 h after transfection for further analyses. To confirm the interaction between BmSPX and BmPSI, the BmE cells were treated with lysis buffer (NP-40, 0.2 mM Phenylmethylsulfonyl fluoride). Lysates were incubated with the anti-Flag affinity gel (Sigma, St Louis, MO, USA) for 12 h at 4 °C. Protein signals were then detected by Western blot using mouse anti-Flag antibody (Beyotime, Shanghai, China) and rabbit anti-Myc antibody (Beyotime, Shanghai, China). The Co-IP groups are shown in Table 4.

### 4.7. Construction of Recombinant Expression Vectors

The pET His_6_ MBP TEV LIC cloning vector is an LIC N-terminal fusion vector for *Escherichia coli* expression, containing a maltose binding protein (MBP). The wild-type *Bmpsi* sequence was cloned into this vector behind a (His)_6_ affinity tag by LIC cloning.

### 4.8. Preparation of Single Stranded RNA

Single-stranded RNA probe CE1 was prepared for EMSA. The RNA probe was labeled with Biotin on its 5′ -end and 3′ -end. The sequence of the single-stranded RNA was the same as the sequence of CE1 in female-specific splicing of *Bmdsx* (5′-uuaauaauauaaguggugua-3′). The RNA probe was compounded by the Beijing Genomics Institute and purified with ion exchange High Performance Liquid Chromatography The RNA was dissolved in a buffer (20 mM Tris, 20 mM NaCl), which was used to purify MBP-BmPSI and MBP protein.

### 4.9. The Overexpression and Purification of MBP-BmPSI and MBP

Transetta (DE3) Chemically Competent Cell was used to overexpress the wild-type BmPSI with the recombinant vectors discussed above. We overexpressed the maltose-binding protein with the original expression vector as a negative control. The purification of target proteins needed 2 L cells in Luria-Bertani culture media at 37 °C with an absorbance at 600 nm of 0.5. Then, we added Isopropyl-beta-D-thiogalactopyranoside to a final concentration of 1 mM and cultured the cells at 37 °C for another 4 h. After cell culturing, the cells were gathered and crushed with multi-gelation three times and ultra-sonicated (40%, 30 min). The supernatant was separated using centrifugation (15,000 g, 10 min). A series of buffers with different concentrations of imidazole were used to wash the HiTrap Chelating where the supernatant was loaded. The buffer containing the target protein was passed through gel filtration chromatography, which made it purer. Finally, we retrieved the pure target protein with the (His)_6_-MBP-TEV component.

### 4.10. Electrophoretic Mobility Shift Assay

Electrophoretic mobility shift assay (EMSA) can be used to determine binding affinity, specific, and stoichiometry of the RNA/protein interaction. In this article, we used the EMSA experiment to verify the interaction between MBP-BmPSI and CE1, with MBP and CE1 as a negative control.

### 4.11. Quantification and Statistical Analysis

All the data are shown as mean ± standard deviation (SD). Statistical analyses were conducted using Microsoft Excel and GraphPad Prism. Two-tailed, paired Student’s t-tests were used to determine statistical significance when comparing two groups. A value of *p* < 0.05 was considered to be statistically significant (* *p* < 0.05, ** *p* < 0.01, *** *p* < 0.001).

## Figures and Tables

**Table 1:** Insertion site of Over-BmSPX strains.

Transgenic Line	Sequence of Insertion Sites	Chromosome (nscaf)
Over-BmSPX	CCATATTGTTTTAA-piggyBac-TTAAAATCTACGAC	11 (16)

**Table 2:** Primer sequences of vector construction.

Name	Primers
*Flag-BmSpx*	5′-cgcggatccatggattacaaggatgacgacgataaggcagcggggcctatt-3′
	5′ataagaatgcggccgctcaataattatagtttgg-3′
*Myc-EGFP*	5′-cgcggatccatggagcagaaactcatctctgaagaggatctggtgagcaagggcgagga-3′
	5′-ataagaatgcggccgcttacttgtacagctcgtccatg-3′
*Flag-EGFP*	5′-cgcggatccatggattacaaggatgacgacgataaggtgagcaagggcgagga-3′
	5′-ataagaatgcggccgcttacttgtacagctcgtccatg-3′
*Myc-BmPsi*	5′-cgcggatccatggagcagaaactcatctctgaagaggatctgagtgattattcttctatggct-3′
	5′-ataagaatgcggccgctcactgctggtggtcggagccggc-3′

**Table 3:** Primer sequences for Real-time PCR.

Name (Gene Acc. No.)	Primers
qPCR-BmSpx (NM_001044181.1)	5′-atcagggctatggatttg-3′, 5′-ccaaatgcagagaatgtg-3′
qPCR-BmMasc (NM_001309577.1)	5′-atggcaaaactggatgacgc-3′, 5′-cccttttgacaccacatgct-3′
qPCR-BmImp (XM_004929851.3 _)	5′-aggcgcagtatcttatctttga-3′, 5′-ccacgacaatttccacaatcag-3′
qPCR-Bmdsx-F (NM_001043406.1)	5′-aaccatgccaccactgataccaac-3′, 5′-gcacaacgaatactgctgcaatcg-3′
qPCR-BmAbd-B (NM_001146228.1)	5′-ctatcctccagatgctcccg-3′, 5′-accctgatgacagcctccat-3′
qPCR-BmTGIF (XM_012688716.2)	5′-cggagctgatgttgagaatg-3′, 5′-accgcactggaggagtagcc-3′
qPCR-Bmspi (XM_004926152.2)	5′-actgtgagtgtcaaagcgggtat-3′, 5′-ggacgcagtctccatcatcag-3′
qPCR-Bmrho(XM_004932433.3)	5′-gagatcggaagtattatcaggagc-3′, 5′-ccaactctaacagtgtaacgcaga-3′
qPCR-Bmcbl (XM_021347118.1)	5′-cgaaaacgacaaggacatcag-3′, 5′-aatcaatttgccacgcagtg-3′
qPCR-Bmhrs (XM_021352179.1)	5′-gaccggaactattgggagca-3′, 5′-gttggaggcagtggaagcag-3′
qPCR-BmcyclinA (NM_001160187.1)	5′-gggagaccacttacaaacctttt-3′, 5′-tcacattttcagcagcagcattcac-3′
qPCR-BmcyclinB (NM_001043878.2)	5′-cgggaaaggtaatggagcc-3′, 5′-gtactacgccacggtttaggg-3′
qPCR-BmcyclinD (NM_001257007.1)	5′-gctccagaggttgaattggc-3′, 5;-agaagttaaggtgagggcgtgt-3′
qPCR-BmcyclinL (NM_001161717.1)	5′-caaaaccaaccgaagtctaacaa-3′, 5′-gagcgtcaaaactatcttcccata-3′

**Table 4:** CO-IP between BmSPX and BmPSI.

Group	Vector 1 (Tag)	Vector 2 (Tag)
Control	pSL1180-BmPSI (Myc)	pSL1180-EGFP (Flag)
Experiment	pSL1180-BmPSI (Myc)	pSL1180-BmSPX (Flag)

## Abbreviations

dsx	Doublesex
tra	Transformer
Masc	Masculinizer
Abd-B	Abdominal-B
SV40	Terminator of *Simian virus 40*
M-MLV	Moloney Murine Leukemia Virus
CDS	Coding sequence
snRNP	Small nuclear ribonucleoprotein
PCR	Polymerase Chain Reaction
MBP	Maltose binding protein
DsRed	*Discosoma* sp. Red Fluorescent Protein
BmE	Embryonic cells of silkworm
PSI	P-element somatic inhibitor
EGFP	Enhanced green fluorescent protein
Co-IP	Co-immunoprecipitation assay
SPX	Spliceosomal protein on the X chromosome
hrs	Hepatocyte growth factor-regulated tyrosine kinase substrate
rho	Rhodopsin
spi	spitz
cbl	Cbl proto-oncogene
TGIF	TGFB-induced factor homeobox
Sxl	Sex lethal
EGFR	Epidermal Growth Factor Receptor
FGFR	Fibroblast Growth Factor Receptor
RTK	Receptor Tyrosine Kinase
qPCR	Quantitative Real-Time PCR
EMSA	Electrophoretic mobility shift assay

## References

[1] Verhulst E.C., van de Zande L., Beukeboom L.W. (2010). Insect sex determination: It all evolves around transformer. Curr. Opin. Genet. Dev..

[2] Suzuki M.G. (2010). Sex determination: Insights from the silkworm. J. Genet..

[3] Cho S., Huang Z.Y., Zhang J. (2007). Sex-specific splicing of the honeybee doublesex gene reveals 300 million years of evolution at the bottom of the insect sex-determination pathway. Genetics.

[4] Salz H.K., Erickson J.W. (2010). Sex determination in Drosophila: The view from the top. Fly.

[5] Shearman D.C. (2002). The evolution of sex determination systems in dipteran insects other than Drosophila. Genetica.

[6] Ohbayashi F., Suzuki M.G., Mita K., Okano K., Shimada T. (2001). A homologue of the Drosophila doublesex gene is transcribed into sex-specific mRNA isoforms in the silkworm, Bombyx mori. Comp. Biochem. Phys. B.

[7] Fujii T., Shimada T. (2007). Sex determination in the silkworm, Bombyx mori: A female determinant on the W chromosome and the sex-determining gene cascade. Semin. Cell Dev. Biol..

[8] Papagiannouli F., Lohmann I. (2015). Stage-specific control of stem cell niche architecture in the Drosophila testis by the posterior Hox gene Abd-B. Comput. Struct. Biotechnol. J..

[9] Ronshaugen M., Levine M. (2004). Visualization of trans-homolog enhancer-promoter interactions at the Abd-B Hox locus in the Drosophila embryo. Dev. Cell.

[10] Wang Y., Zhao Q., Wan Q.X., Wang K.X., Zha X.F. (2019). P-element Somatic Inhibitor Protein Binding a Target Sequence in dsx Pre-mRNA Conserved in Bombyx mori and Spodoptera litura. Int. J. Mol. Sci..

[11] Suzuki M.G., Ohbayashi F., Mita K., Shimada T. (2001). The mechanism of sex-specific splicing at the doublesex gene is different between Drosophila melanogaster and Bombyx mori. Insect Biochem. Mol. Biol..

[12] Suzuki M.G., Imanishi S., Dohmae N., Nishimura T., Shimada T., Matsumoto S. (2008). Establishment of a novel in vivo sex-specific splicing assay system to identify a trans-acting factor that negatively regulates splicing of Bombyx mori dsx female exons. Mol. Cell. Biol..

[13] Xu J., Chen S., Zeng B., James A.A., Tan A., Huang Y. (2017). Bombyx mori P-element Somatic Inhibitor (BmPSI) Is a Key Auxiliary Factor for Silkworm Male Sex Determination. PLoS. Genet..

[14] Suzuki M.G., Kobayashi S., Aoki F. (2014). Male-specific splicing of the silkworm Imp gene is maintained by an autoregulatory mechanism. Mech. Dev..

[15] Katsuma S., Sugano Y., Kiuchi T., Shimada T. (2015). Two Conserved Cysteine Residues Are Required for the Masculinizing Activity of the Silkworm Masc Protein. J. Biol. Chem..

[16] Shimada T. (2004). [Sex determination mechanism in the silkworm, Bombyx mori, compared with that in Drosophila]. Tanpakushitsu Kakusan Koso..

[17] Kawaoka S., Kadota K., Arai Y., Suzuki Y., Fujii T., Abe H., Yasukochi Y., Mita K., Sugano S., Shimizu K. (2011). The silkworm W chromosome is a source of female-enriched piRNAs. RNA.

[18] Kiuchi T., Koga H., Kawamoto M., Shoji K., Sakai H., Arai Y., Ishihara G., Kawaoka S., Sugano S., Shimada T. (2014). A single female-specific piRNA is the primary determiner of sex in the silkworm. Nature.

[19] Hara K., Fujii T., Suzuki Y., Sugano S., Shimada T., Katsuma S., Kawaoka S. (2012). Altered expression of testis-specific genes, piRNAs, and transposons in the silkworm ovary masculinized by a W chromosome mutation. BMC Genom..

[20] Zhao Q., Li J., Wen M.Y., Wang H., Wang Y., Wang K.X., Wan Q.X., Zha X.F. (2019). A Novel Splice Variant of the Masculinizing Gene Masc with piRNA-Cleavage-Site Defect Functions in Female External Genital Development in the Silkworm, Bombyx mori. Biomolecules.

[21] Zheng Z.-Z., Sun X., Zhang B., Pu J., Jiang Z.-Y., Li M., Fan Y.-J., Xu Y.-Z. (2019). Alternative splicing regulation of doublesex gene by RNA-binding proteins in the silkworm Bombyx mori. RNA Biol..

[22] Zha X.F., Zhao M., Zhou C.Y., Guo H.Z., Zhao P., Xiang Z.H., Xia Q.Y. (2014). Analysis of interaction between Bmhrp28 and BmPSI in sex-specific splicing of Bombyx mori Bmdsx gene. Genet. Mol. Res..

[23] Devotta A., Juraver-Geslin H., Gonzalez J.A., Hong C.S., Saint-Jeannet J.P. (2016). Sf3b4-depleted Xenopus embryos: A model to study the pathogenesis of craniofacial defects in Nager syndrome. Dev. Biol..

[24] Bernier F.P., Caluseriu O., Ng S., Schwartzentruber J., Buckingham K.J., Innes A.M., Jabs E.W., Innis J.W., Schuette J.L., Gorski J.L. (2012). Haploinsufficiency of SF3B4, a component of the pre-mRNA spliceosomal complex, causes Nager syndrome. Am. J. Hum. Genet..

[25] Zhang P., Cao G., Sheng J., Xue R., Gong C. (2012). BmTGIF, a Bombyx mori homolog of Drosophila DmTGIF, regulates progression of spermatogenesis. PLoS ONE.

[26] Papagiannouli F., Schardt L., Grajcarek J., Ha N., Lohmann I. (2014). The Hox gene Abd-B controls stem cell niche function in the Drosophila testis. Dev. Cell.

[27] Casali A., Casanova J. (2001). The spatial control of Torso RTK activation: A C-terminal fragment of the Trunk protein acts as a signal for Torso receptor in the Drosophila embryo. Development.

[28] Tsai H.F., Huang C.W., Chang H.F., Chen J.J., Lee C.H., Cheng J.Y. (2013). Evaluation of EGFR and RTK signaling in the electrotaxis of lung adenocarcinoma cells under direct-current electric field stimulation. PLoS ONE.

[29] Tang X.F., Zhou X.L., Zhang Q., Chen P., Lu C., Pan M.H. (2018). Bombyx mori cyclin-dependent kinase inhibitor is involved in regulation of the silkworm cell cycle. Insect Mol. Biol..

[30] Salvaing J., Mouchel-Vielh E., Bloyer S., Preiss A., Peronnet F. (2008). Regulation of Abd-B expression by Cyclin G and Corto in the abdominal epithelium of Drosophila. Hereditas.

[31] Wang K., Yin C., Du X., Chen S., Wang J., Zhang L., Wang L., Yu Y., Chi B., Shi M. (2019). A U2-snRNP-independent role of SF3b in promoting mRNA export. Proc. Natl. Acad. Sci. USA.

[32] Martelly W., Fellows B., Senior K., Marlowe T., Sharma S. (2019). Identification of a noncanonical RNA binding domain in the U2 snRNP protein SF3A1. RNA.

[33] van der Feltz C., Hoskins A.A. (2019). Structural and functional modularity of the U2 snRNP in pre-mRNA splicing. Crit. Rev. Biochem. Mol. Biol..

[34] Jiang L., Cheng T., Zhao P., Yang Q., Wang G., Jin S., Lin P., Xiao Y., Xia Q. (2012). Resistance to BmNPV via overexpression of an exogenous gene controlled by an inducible promoter and enhancer in transgenic silkworm, *Bombyx mori*. PloS ONE.

